# Erythropoiesis-stimulating agent resistance and mortality in hemodialysis and peritoneal dialysis patients

**DOI:** 10.1186/1471-2369-14-200

**Published:** 2013-09-25

**Authors:** Marit M Suttorp, Tiny Hoekstra, Joris I Rotmans, Ilka Ott, Moshe Mittelman, Raymond T Krediet, Friedo W Dekker

**Affiliations:** 1 Department of Clinical Epidemiology, Leiden University Medical Center, Leiden, The Netherlands; 2 Department of Nephrology, Leiden University Medical Center, Leiden, The Netherlands; 3 Deutsches Herzzentrum der Technischen Universität München, München, Germany; 4 Department of Medicine, Tel Aviv Sourasky Medical Center, Sackler Faculty of Medicine, Tel Aviv, Israel; 5 Department of Nephrology, Academic Medical Center, University of Amsterdam, Amsterdam, The Netherlands

**Keywords:** Cohort study, Erythropoietin, ESA Resistance, Hemodialysis, Mortality, Peritoneal dialysis

## Abstract

**Background:**

Responsiveness to erythropoiesis-stimulating agents (ESAs) varies widely among dialysis patients. ESA resistance has been associated with mortality in hemodialysis (HD) patients, but in peritoneal dialysis (PD) patients data is limited. Therefore we assessed the relation between ESA resistance in both HD and PD patients.

**Methods:**

NECOSAD is a Dutch multi-center prospective cohort study of incident dialysis patients who started dialysis between January 1997 and January 2007. ESA resistance was defined as hemoglobin level < 11 g/dL with an above median ESA dose (i.e. 8,000 units/week in HD and 4,000 units/week in PD patients). Unadjusted and adjusted Cox regression analysis for all-cause 5-year mortality was performed for HD and PD patients separately.

**Results:**

1013 HD and 461 PD patients were included in the analysis. ESA resistant HD patients had an adjusted hazard ratio of 1.37 (95% CI 1.04-1.80) and ESA resistant PD patients had an adjusted hazard ratio of 2.41 (1.27-4.57) as compared to patients with a good response.

**Conclusions:**

ESA resistance, as defined by categories of ESA and Hb, is associated with increased mortality in both HD and PD patients. The effect of ESA resistance, ESA dose and hemoglobin are closely related and the exact mechanism remains unclear. Our results strengthen the need to investigate and treat causes of ESA resistance not only in HD, but also in PD patients.

## Background

Anemia is a common complication of end stage renal disease and has been associated with increased mortality in dialysis patients [1]. Therefore about 90% of dialysis patients receive erythropoiesis-stimulating agents (ESAs) [2]. ESAs are prescribed and titrated according to the change in hemoglobin (Hb) level, but responsiveness to ESAs varies widely among dialysis patients [3]. The patients with a reduced hematopoietic response to ESAs are called ESA resistant or hyporesponsive [4,5].

Different definitions are being used to define ESA resistance, based on Hb change per ESA dose increase, categories of ESA dose and Hb or erythropoietin resistance index (ERI) [6-8]. The KDOQI guideline describes ESA resistance as an inappropriately low Hb for the administered ESA dose, which includes patients who fail to achieve the target Hb of 11 g/dL while treated with high ESA doses [9].

ESA resistance has been associated with a 1.4- to a more than 2-fold increased mortality rate [10-13]. This association has predominantly been studied in hemodialysis (HD) patients, whereas 20 to 30% of dialysis patients in the Netherlands are treated with peritoneal dialysis (PD) [14]. Only one previous US study examined the association between ESA resistance and mortality separately for HD and PD patients, in which ESA resistance was associated with higher mortality rates in HD patients. However, this was not confirmed in PD patients [15]. Only PD patients treated with excessively high ESA doses had a higher mortality rate. To verify the different association between ESA resistance and mortality in HD and PD patients, these results need replication, especially in a European patient population with lower ESA doses.

Therefore, we aimed to assess the association between ESA resistance and mortality during 5 years of follow-up in both HD and PD patients. In a prospective cohort of incident dialysis patients in the Netherlands, we identified ESA resistant patients by categories of ESA dose and Hb level.

## Methods

### Study design and participants

The Netherlands Cooperative Study on the Adequacy of Dialysis (NECOSAD-II) is a prospective cohort study in which incident dialysis patients from 38 dialysis centers throughout the Netherlands were enrolled between January 1997 and January 2007. Patients were eligible if they started dialysis for the first time and were 18 years or older. All patients gave written informed consent before inclusion. Baseline was defined as 180 days after start of dialysis to account for the first period with therapy switches and deaths probably due to health status before start of dialysis. Furthermore, at this time point 92% of the ESA using dialysis patients were treated with ESAs for at least 3 months and ESA dose and Hb can be considered in a stable phase. The NECOSAD study was approved by the medical ethical committees of all participating centers (Maasstad Hospital Rotterdam, Deventer Hospital Deventer, Sint Lucas Andreas Hospital Amsterdam, Academic Medical Center Amsterdam, Maxima Medical Center Veldhoven, Catharina Hospital Eindhoven, Medical Center Haaglanden Den Haag, University Medical Center Groningen, Kennemer Gasthuis Haarlem, Atrium Medical Center Heerlen, Medical Center Leeuwarden, Leiden University Medical Center Leiden, Elisabeth Hospital Tilburg, University Medical Center Utrecht, Antonius Hospital Nieuwegein, Hospital Gelderse Vallei Ede, Haga Hospital Leyenburg Den Haag, Academic Hospital Maastricht, Jeroen Bosch Hospital Den Bosch, Medisch Spectrum Twente Enschede, Albert Schweitzer Hospital Dordrecht, Alysis Zorggroep Rijnstate Hospital Arnhem, Dianet Dialysis Center Lunetten Utrecht, Canisius Wilhelmina Hospital Nijmegen, Vie Curi Medical Center Venlo, Leveste Scheper Hospital Emmen, Dianet Dialysis Center Holendrecht Amsterdam, Haga Hospital Rode Kruis Den Haag, Rijnland Hospital Leiderdorp, Admiraal de Ruyter Hospital Goes, Medical Center Alkmaar, Laurentius Hospital Roermond, Dialysis Center ’t Gooi Hilversum, Groene Hart Hospital Gouda, Westfries Gasthuis Hoorn, Tergooi Hospitals Hilversum, Martini Hospital Groningen, Zaans Medical Center Zaandam). For the present analysis, all patients treated with ESAs and with data on Hb and ESA dose at 6 months after start of dialysis were included.

### Patient characteristics

Patient characteristics were collected at start of dialysis, including age, gender and primary kidney disease. Primary kidney disease was classified according to the European Renal Association- European Dialysis and Transplant Association (ERA-EDTA) codes [16]. Data on weight, comorbidity and laboratory parameters were collected six months after start of dialysis. Comorbid conditions were classified by the nephrologist. Cardiovascular disease consisted of angina pectoris, myocardial infarction, heart failure, stroke and claudication. Nutritional status was measured by trained nurses with the 7-point subjective global assessment (SGA) [17]. A score of 1 or 2 was classified as severely malnourished and 6 or 7 as well-nourished. For HD patients, blood samples were taken before the dialysis session. Hb, albumin, ferritin, parathyroid hormone (PTH), creatinine, urea and C-reactive protein (CRP) were measured in serum. Urea and creatinine were also determined in urine. Residual glomerular filtration rate (rGFR) was calculated as the mean of creatinine and urea clearance corrected for body surface area (mL/min per 1.73 m^2^). Dialysis dose was expressed as total Kt/V urea per week and calculated according to Watson et al. [18].

### ESA resistance

ESA dose was registered in units per week at baseline. Darbepoetin dose in micrograms was converted to units by multiplying with 200. Patients were divided into four categories based on target Hb and median ESA dose. Patients were considered ESA resistant if they failed to reach target Hb (11 g/dL) [9,19] while treated with an above median ESA dose (8,000 units/week for HD and 4,000 units/week for PD patients). Good ESA responders reached target Hb with a median or lower ESA dose.

### Outcome definition

The endpoint was 5-year mortality. Cause of death was classified according to the ERA-EDTA codes [19]. Cardiovascular mortality was defined as death due to myocardial ischemia and infarction, hyperkalemia, hypokalemia, cardiac arrest, cardiac failure, fluid overload, cerebrovascular accident, pulmonary embolism, hemorrhage from ruptured aneurysm, mesenteric infarction and cause of death uncertain.

### Statistical analyses

Continuous variables are presented as mean with standard deviation (SD) or median with interquartile range (IQR), depending on the normality of the data. Categorical variables are presented as percentages. Survival time was calculated as time between baseline and date of death or censoring (i.e. renal transplantation, recovery of renal function, withdrawal of participation or end of follow-up in May 2009). All analyses were stratified by modality. Mortality rates were calculated and Cox regression analysis was used to calculate hazard ratios (HR) with 95% confidence interval (CI) for 5-year all-cause mortality. Patients with a good ESA response served as the reference category. The proportional hazards assumption was verified by plotting the log minus log plots. Analyses were adjusted for a priori defined confounders age, sex, primary kidney disease, cardiovascular disease, malignancy, diabetes mellitus, Kt/Vurea, rGFR, albumin, ferritin, PTH and CRP. Missings were less than 3%, except for PTH (4.6%), albumin (5.3%), SGA (15.6%), rGFR (22.0%), Kt/Vurea (23.4%) and CRP (51.2%). Missing data were imputed with standard multiple imputation techniques with 10 repetitions, which are based on the Markov Chain Monte Carlo (MCMC) method [20]. Results for cardiovascular and non-cardiovascular causes of death were also analysed separately.

### Sensitivity analysis

To explore dose dependent effects of ESA and Hb on mortality, analysis with more categories of ESA and Hb were conducted. Furthermore, ERI was calculated by dividing the weekly ESA dose per kilogram by Hb (weekly units/kg/Hb). Patients were grouped into quartiles of ERI, in which the highest ERI quartile corresponds with ESA resistance. Cox regression analyses were performed with the lowest ERI quartile as a reference. Last, since CRP was missing in a high percentage of patients, we also performed our analyses only in those patients with a measured CRP and without adjustment for CRP, to further validate our results. All analyses were performed with SPSS statistical software version 20.0.

## Results

A total of 2051 patients were enrolled and after 6 months 1791 patients were still on dialysis, 34 received kidney transplantation, 140 died, 19 experienced recovery of renal function, 52 withdrew participation and 15 patients left for other reasons. Of the 1791 patients 1528 were treated with ESA. Data on Hb and ESA dose were available in 1474 patients (1013 HD and 461 PD) and those patients were included in the present analysis. Median ESA dose was 8,000 units/week in HD and 4,000 units/week in PD patients.

In Table 1 and Table 2 baseline characteristics of HD and PD patients are shown by ESA and Hb categories. PD patients were younger than HD patients and rGFR was higher in PD patients. Overall, ESA resistant patients had lower rGFR, lower albumin levels and higher ferritin levels. PD patients with higher ESA doses had more comorbidities and higher CRP in general, while for HD the comorbidities and higher CRP were more pronounced in ESA resistant patients.

**Table 1:** Baseline characteristics of HD patients

**HD patients**	**ESA ≤8,000 (units/week)**		**ESA >8,000 (units/week)**	
	**Hb ≥11 (g/dl)**	**Hb <11 (g/dl)**	**Hb ≥11 (g/dl)**	**Hb <11 (g/dl)**
**Number**	**380**	**264**	**158**	**211**
Age (years)	64.5 (13.2)	64.1 (14.4)	64.9 (13.7)	64.2 (12.8)
Sex (%) men	60.4	56.1	65.0	56.4
Weight (kg)	70.6 (12.4)	72.0 (14.3)	74.1 (14.9)	73.6 (14.8)
ESA dose (units/week)	6,000 (4,000-8,000)	6,000 (4,000-8,000)	12,000 (10,000-16,000)	12,000 (12,000-18,000)
ERI (units/week/kg/Hb)	6.5 (2.9)	7.9 (3.4)	16.0 (6.3)	22.3 (13.5)
Primary kidney disease (%)				
- Diabetes mellitus	10.6	17.4	16.5	15.6
- Glomerulonephritis	11.1	7.6	8.2	8.5
- Renal vascular disease	22.2	21.6	12.7	19.0
- Other	56.2	53.4	62.7	56.9
Comorbidity (%)				
- Diabetes mellitus	17.1	20.8	27.2	24.2
- Malignancy	7.4	9.8	11.4	18.5
- Cardiovascular disease	39.2	41.3	32.9	40.8
Weekly Kt/V urea (Watson)	3.7 (0.9)	3.5 (0.9)	3.7 (0.8)	3.7 (1.0)
rGFR (ml/min/1.73 m^2^)	2.9 (1.5-4.4)	2.3 (0.8-3.8)	2.1 (0.8-4.2)	1.5 (0.4-3.2)
Nutritional status (SGA) (%)				
- Severely malnourished	0.3	2.2	0.8	5.4
- Mild-moderate	19.1	25.4	27.1	37.3
- Well-nourished	80.6	72.4	72.0	57.2
Laboratory values				
- Hemoglobin (g/dl)	12.1 (11.6-12.7)	10.3 (9.5-10.6)	12.1 (11.6-12.8)	9.8 (9.2-10.5)
- Albumin (g/l)	37.5 (4.5)	36.0 (4.9)	35.6 (4.5)	34.1 (6.0)
- Ferritin (μg/l)	190 (104–380)	175 (94–379)	229 (121–434)	257 (118–511)
- PTH (pmol/l)	12.1 (5.5-23.8)	10.6 (4.7-23.9)	16.3 (6.4-33.7)	10.0 (3.2-23.4)
- CRP (mg/l)	6.0 (3.0-12.0)	6.0 (3.0-15.0)	9.0 (3.0-17.0)	14.0 (6.0-34.5)

Values are presented as mean (SD), median (IQR) or percentage.

**Table 2:** Baseline characteristics of PD patients

**PD patients**	**ESA ≤4,000 (units/week)**		**ESA >4,000 (units/week)**	
	**Hb ≥11 (g/dl)**	**Hb <11 (g/dl)**	**Hb ≥11 (g/dl)**	**Hb <11 (g/dl)**
**Number**	**204**	**91**	**113**	**53**
Age (years)	52.0 (14.7)	50.6 (15.1)	57.9 (15.5)	49.8 (16.1)
Sex (%) men	64.2	65.9	73.2	60.4
Weight (kg)	73.1 (12.8)	73.5 (14.3)	77.8 (13.7)	77.9 (16.8)
ESA dose (units/week)	4,000 (2,000-4,000)	4,000 (2,000-4,000)	8,000 (6,000-8,000)	9,000 (8,000-12,000)
ERI (units/week/kg/Hb)	3.4 (1.4)	4.5 (1.8)	9.0 (4.4)	14.9 (8.5)
Primary kidney disease (%)				
- Diabetes mellitus	10.8	19.8	17.0	13.2
- Glomerulonephritis	18.1	17.6	17.9	17.0
- Renal vascular disease	7.8	8.8	21.4	13.2
- Other	63.2	53.8	43.8	56.6
Comorbidity (%)				
- Diabetes mellitus	13.2	19.8	23.9	15.1
- Malignancy	5.4	2.2	8.8	5.7
- Cardiovascular disease	19.6	17.6	31.9	20.8
Weekly Kt/V urea (Watson)	2.3 (0.6)	2.1 (0.4)	2.3 (0.6)	2.1 (0.5)
rGFR (ml/min/1.73 m^2^)	3.7 (2.0-5.6)	2.7 (1.0-3.8)	3.1 (1.2-5.1)	1.3 (0.5-3.3)
Nutritional status (SGA) (%)				
- Severely malnourished	0.6	0.0	0.0	0.0
- Mild-moderate	13.4	24.7	15.6	22.7
- Well-nourished	86.0	75.3	84.4	77.3
Laboratory values				
- Hemoglobin (g/dl)	12.4 (11.8-13.3)	9.7 (10.3-10.8)	12.4 (11.7-13.2)	10.0 (9.5-10.8)
- Albumin (g/l)	37.4 (4.7)	35.4 (6.0)	36.0 (5.5)	34.0 (6.1)
- Ferritin (μg/l)	138 (63–232)	186 (81–294)	99 (51–180)	218 (114–479)
- PTH (pmol/l)	11.6 (4.6-23.8)	10.8 (3.6-31.3)	12.1 (4.3-28.9)	16.4 (3.5-25.0)
- CRP (mg/l)	4.0 (3.0-8.0)	4.0 (3.0-14.0)	9.5 (5.0-16.3)	7.0 (3.0-11.0)

Values are presented as mean (SD), median (IQR) or percentage.

### Outcomes

A total of 572 patients died during 5 years of follow-up. The observed rates of all-cause mortality are listed in Table 3. In both HD and PD patients the mortality rate was around twice as high in the ESA resistant group as compared to the good responders. In PD the mortality rate in patients with a high ESA dose but with a good Hb response was somewhat higher than in the ESA resistant patients. The highest all-cause mortality was observed in ESA resistant HD patients (243 deaths per 1,000 person years).

**Table 3:** Mortality rates for all-cause mortality by ESA and Hb category

			**All-cause mortality**		
**ESA (units/week)**	**Hb (g/dl)**	**Person years**	**Death (n)**	**Death rate/1,000 person years**	**95% CI**
• **HD patients**					
≤8,000	≥11	1002	142	142	(120–167)
≤8,000	<11	661	121	183	(153–218)
>8,000	≥11	358	68	190	(149–239)
>8,000	<11	469	114	243	(202–291)
• **PD patients**					
≤4,000	≥11	527	39	74	(53–100)
≤4,000	<11	218	25	115	(76–167)
>4,000	≥11	247	45	182	(134–241)
>4,000	<11	109	18	166	(101–257)

### ESA resistance and mortality

The unadjusted HR for ESA resistant HD patients as compared to good responders according to ESA and Hb categories was 1.72 (95% CI 1.34-2.20) as shown in Table 4. After adjustment for general characteristics and comorbidities ESA resistance was still associated with an almost 2 fold increased all-cause mortality rate. Full adjustment downgraded the HR to 1.37 (95% CI 1.04-1.80). In PD patients both groups with high ESA doses were associated with increased mortality in crude analysis, irrespective of Hb. After full adjustment the HR in ESA resistant PD patients was 2.41 (95% CI 1.27-4.57). The higher mortality rate in PD patients with high ESA dose but with good Hb attenuated mainly after adjustment for co-morbidities, resulting in a fully adjusted HR of 1.56 (95% CI 0.96-2.51).

**Table 4:** Hazard ratios for all-cause mortality by ESA and Hb category

				**All-cause mortality**	
**ESA (units/week)**	**Hb (g/dL)**	**Patients (n)**	**Unadjusted HR**	** Adjusted HR**^**1**^	** Adjusted HR**^**2**^
• **HD patients**					
≤8,000	≥11	380	1	1	1
≤8,000	<11	264	1.29 (1.01-1.65)	1.33 (1.04-1.70)	1.18 (0.91-1.52)
>8,000	≥11	158	1.35 (1.01-1.80)	1.52 (1.13-2.04)	1.28 (0.94-1.73)
>8,000	<11	211	1.72 (1.34-2.20)	1.91 (1.48-2.47)	1.37 (1.04-1.80)
• **PD patients**					
≤4,000	≥11	204	1	1	1
≤4,000	<11	91	1.59 (0.96-2.63)	1.94 (1.15-3.27)	1.56 (0.91-2.68)
>4,000	≥11	113	2.55 (1.66-3.92)	1.89 (1.20-2.97)	1.56 (0.96-2.51)
>4,000	<11	53	2.34 (1.34-4.10)	3.18 (1.74-5.79)	2.41 (1.27-4.57)

Values are shown as hazard ratios (95% CI).

Categories are defined by a combination of ESA dose (below or from median) and Hb level (≥11 and <11 g/dL). The category with the high ESA dose and Hb <11 corresponds to ESA resistant patients and the category with the low ESA dose and Hb ≥11 corresponds to good ESA responders.

^1^ Adjusted for age, sex, weight, primary kidney disease, diabetes mellitus, malignancy, cardiovascular disease.

^2^ Additional adjusted for weekly Kt/V urea, rGFR, nutritional status, albumin, ferritin, PTH and CRP.

### Causes of death

**Figure 1:**
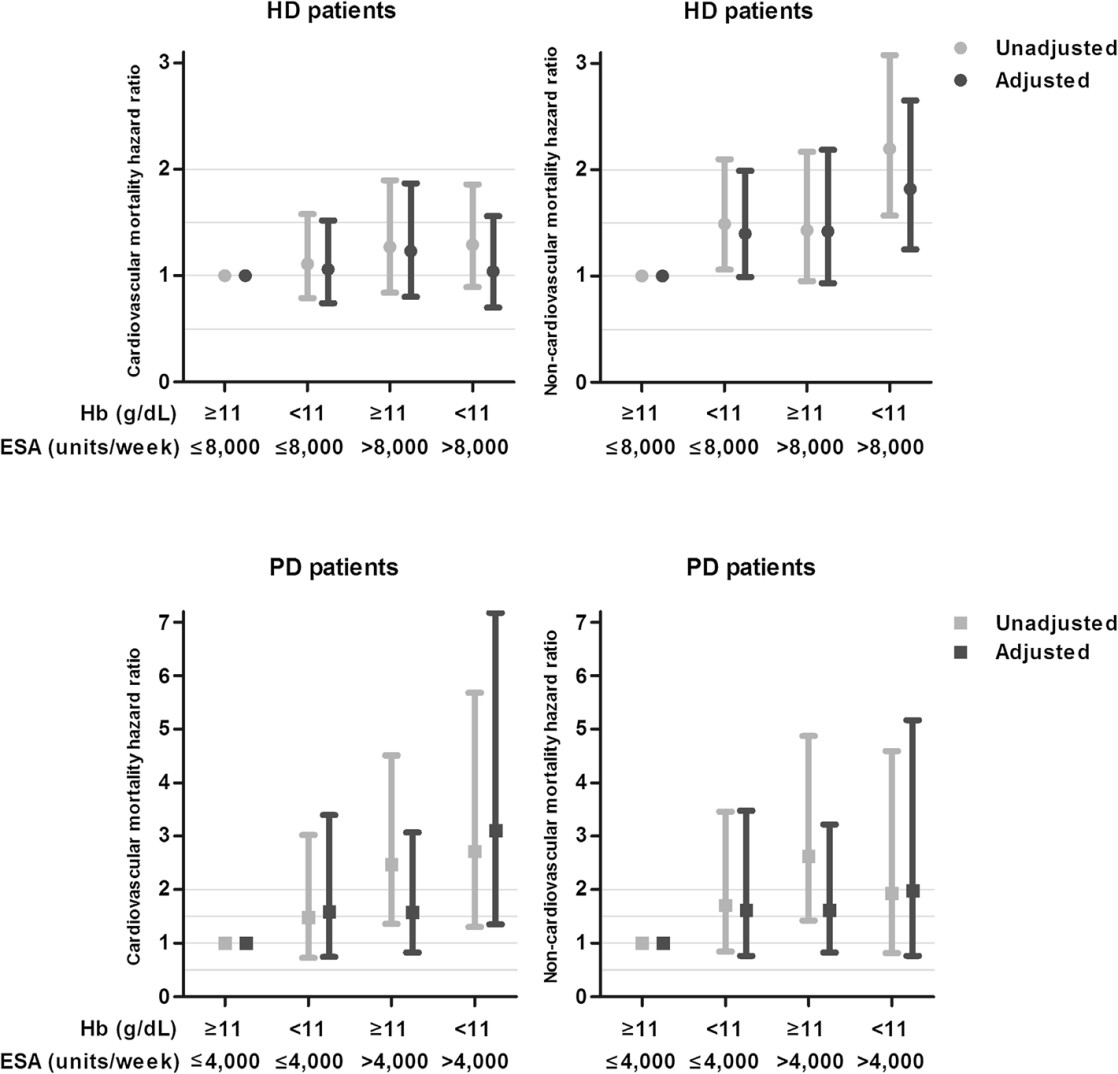
**Hazard ratios for cardiovascular and non-cardiovascular mortality by ESA and Hb category.** Values are shown as hazard ratios (95% Confidence intervals). Adjusted for age, sex, weight, primary kidney disease, diabetes mellitus, malignancy, cardiovascular disease, weekly Kt/V urea, rGFR, nutritional status, albumin, ferritin, PTH and CRP.

In HD patients the relation of ESA resistance with non-cardiovascular mortality was more evident than with cardiovascular mortality (Figure 1). The HR for non-cardiovascular mortality was 1.82 (95% CI 1.25-2.65) and cardiovascular mortality was 1.04 (95% CI 0.70-1.56). In PD patients ESA resistance was associated with both causes of death, although the association with cardiovascular mortality was more pronounced and statistically significant. The adjusted hazard ratio for cardiovascular mortality was 3.11 (95% CI 1.35-7.18) and for non-cardiovascular mortality 1.98 (95% CI 0.76-5.17). Analyses based on ERI quartiles did not materially change these results.

### Sensitivity analyses

**Figure 2:**
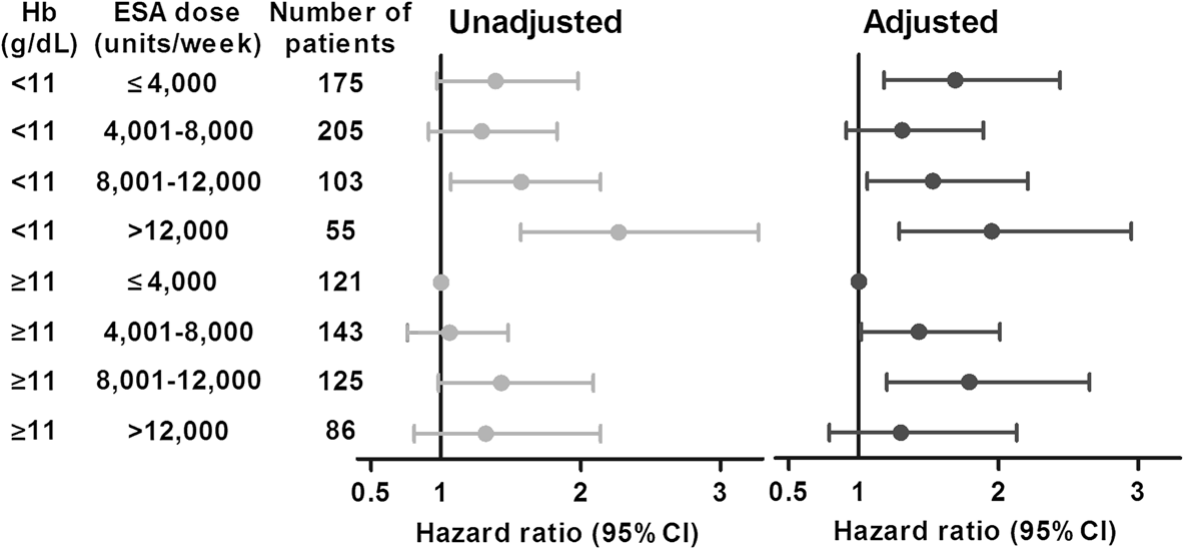
**All-cause mortality for 8 categories of ESA and Hb in HD patients.** Values are shown as hazard ratios (95% Confidence intervals). Adjusted for age, sex, weight, primary kidney disease, diabetes mellitus, malignancy, cardiovascular disease, weekly Kt/V urea, rGFR, nutritional status, albumin, ferritin PTH and CRP.

To explore ESA dose dependency, analysis was performed by creating 8 categories of ESA and Hb in HD patients (Figure 2). The number of patients prevented us from creating more categories and performing this analysis in PD patients. Although confidence intervals were wide, point estimate for mortality was highest in the patients with the lowest Hb and highest ESA dose, representing ESA resistant patients. A dose dependent effect of ESA was not definite. In HD patients with a Hb ≥11 g/dL the HR did not further increase in patients with the highest ESA dose. Also, although patients with a low Hb and relatively high ESA doses had increasing mortality rates, the patients with the lowest ESA dose did not show the lowest mortality rate.

In addition, Cox regression analyses were performed with ESA resistance defined as the upper quartile of ERI (Table 5). In HD patients results were in line with the results based on ESA and Hb categories. A dose response relation was observed, although less pronounced after full adjustment, and the mortality rate in the highest ERI quartile remained increased (HR of 1.34 (1.00-1.80)). In PD patients the association between ERI and mortality became more J-shaped. Patients in the lowest ERI quartile had a higher mortality rate than in the middle quartiles. In quartile 2 to 4 mortality rate rose gradually with a slightly increased mortality rate in the ESA resistant patients compared to the first quartile (1.18 (95% CI 0.68-2.07)).

**Table 5:** Hazard ratios for all-cause mortality by ERI quartile

				**All-cause mortality**	
**ERI ESA/kg/Hb**	**ERI quartile**	**Patients (n)**	**Unadjusted HR**	** Adjusted HR**^**1**^	** Adjusted HR**^**2**^
• **HD patients**					
<5.87	1	253	1	1	1
5.87-9.46	2	253	1.08 (0.82-1.43)	1.16 (0.88-1.55)	1.11 (0.83-1.49)
9.47-14.28	3	254	1.33 (1.01-1.74)	1.28 (0.97-1.68)	1.07 (0.81-1.43)
>14.28	4	253	1.81 (1.39-2.36)	1.85 (1.41-2.42)	1.34 (1.00-1.80)
• **PD patients**					
<3.21	1	115	1	1	1
3.21-4.81	2	115	0.63 (0.36-1.12)	0.45 (0.25-0.80)	0.45 (0.25-0.83)
4.82-7.90	3	115	1.24 (0.75-2.03)	0.95 (0.56-1.61)	0.77 (0.43-1.36)
>7.90	4	116	1.72 (1.07-2.77)	1.46 (0.88-2.41)	1.18 (0.68-2.07)

Values are shown as hazard ratios (95% CI).

Categories are defined by quartiles of ERI (weekly ESA dose/kg/Hb). The highest ERI quartile (quartile 4) corresponds to ESA resistant patients and the lowest ERI quartile (quartile 1) corresponds to good ESA responders.

^1^ Adjusted for age, sex, weight, primary kidney disease, diabetes mellitus, malignancy, cardiovascular disease.

^2^ Additional adjusted for weekly Kt/V urea, rGFR, nutritional status, albumin, ferritin, PTH and CRP.

Because of the high percentage of imputed CRP data, our analysis was also repeated only in those patients with known CRP value and without additional adjustment for CRP in all patients. With both analyses our results did not materially change (data not shown).

## Discussion

In this prospective study of 1474 incident dialysis patients, we found that ESA resistance, defined according to categories of ESA and Hb, was associated with an increased 5-year mortality rate as compared to patients with a good response in both HD and PD patients. After adjustment for potential confounders ESA resistance was associated with a more than 2-fold increased mortality rate in PD patients and a 1.4-fold increased mortality rate in HD patients.

Data in PD patients are limited and only one previous US study, in which ESA dose was used to indicate ESA resistance, examined the association between ESA resistance and mortality in both PD and HD patients [15]. In contrast to the analysis with ESA and Hb categories in our study, mortality rate was not increased in ESA resistant PD patients with ESA doses up to 10,000 units/week. Only PD patients with ESA doses exceeding 15,000 units/week, which is considerably higher than in Dutch clinical practice, had a 28% higher mortality rate. Reasons for the discrepant findings could be the dissimilarities in ESA prescribing pattern between the two countries and differences in patient populations. The US study comprised prevalent patients whereas our study included incident dialysis patients. Prevalent PD patients represent healthier patients, because patients who already died or switched to HD because of comorbidities are not taken into account.

Our results in HD patients are in line with other studies relating ESA resistance with mortality. Studies using change in hematocrit [10] or Hb [13] over time report 1.5- to more than 2-fold increased mortality in ESA resistant HD patients. A large study in prevalent HD patients from Japan also defined ESA resistance based on categories of ESA and Hb [8]. They showed that patients with an ESA dose from 6,000 units/week and Hb below 10 g/dL had a HR of 1.94 for all-cause mortality. An Italian study found a 62% increase in mortality rate and 43% increase in cardiovascular events for ESA resistant patients [7].

In literature, different definitions for ESA resistance have been used. In our analyses both ERI quartiles and categories of ESA and Hb identified a group of patients with the worst survival and results were comparable in HD patients. In PD patients however, results with ERI quartiles were less pronounced. In general, we believe ERI is difficult to interpret because dose of ESA is not normally distributed and the relation between ESA dose and Hb is not linear. High ESA doses therefore result in high ERI, irrespective of achieved Hb. For instance, almost all HD patients in the highest ERI quartile in our population are treated with an ESA dose ≥ 10,000 units/week. Hb levels, however, range from below 8 g/dL to above 12 g/dL in these patients. ERI is therefore, to our opinion, more a reflection of ESA dose than of ESA resistance. Identifying ESA resistant patients by ESA dose and Hb level seems more transparent and therefore better interpretable and applicable in clinical practice.

The J-shaped relation in our sensitivity analyses between ERI quartiles and mortality in PD patients is due to a higher mortality rate in the lowest ERI quartile as compared to the middle ERI quartiles. Potential explanations are speculative, but it might at least partly be explained by the definition of the ERI quartiles. More than half of the PD patients with an Hb ≥11 g/dL and a low ESA dose, in our view patients with a good ESA response, are not allocated to the lowest ERI quartile. As a consequence, patients with a good response to ESA are allocated to different ERI quartiles. Also, a quarter of PD patients in the lowest ERI quartile did not reach target Hb level of 11 g/dL and their response to ESA is therefore at least questionable.

In our study HD patients were treated with higher ESA doses than PD patients, which is in agreement with several other studies [15,21]. The lower ESA requirements could be due to a better preservation of residual renal function in PD patients, which results in higher endogenous erythropoietin production. rGFR was indeed higher in PD patients, as was already reported in a previous analysis of this cohort [22]. Alternatively, it has also been speculated that uremic toxins responsible for the inhibition of erythropoiesis could be better removed by a greater clearance of middle molecular weight substances by PD, resulting in a better hematopoietic response [23]. Route of ESA administration could also affect ESA dose and resistance. Various studies have shown that ESA dose can be reduced when patients switch from intravenous to subcutaneous administration, although for darbepoetin with a longer half-life this is not established [24]. In our cohort, route of ESA administration was documented in just 174 patients, in which 98% of PD and only 66% of HD patients received ESA subcutaneously. Data on type of ESA was unavailable. Last, HD patients experience more blood loss because of frequent blood testing, loss in the extracorporal circuit and oozing from vascular access site. Blood loss can cause iron deficiency, resulting in a decreased hematopoietic response and therefore higher ESA requirements.

Indeed, the relatively low ferritin levels in HD and PD patients could indicate that part of the ESA resistance might be caused by iron deficiency. However, ferritin was highest in both ESA resistant HD and PD patients. This might indicate that especially in these patients extra efforts were made to adequately supplement the relative iron deficiency and overcome ESA resistance. On the other hand, since ferritin is also an acute phase protein, it could also reflect the higher inflammation burden in these patients. Because further specifications on iron status are lacking, a residual effect of iron deficiency on ESA resistance cannot be excluded.

Over the last decade several papers have also suggested that ESA administration to cancer patients is associated with increased mortality [25,26], however the topic has remained controversial [27,28]. In renal patients it is not sure whether it is the need to administer high doses of ESA, thus ESA resistance, or the high ESA doses themselves that result in a higher mortality rate. Anemia correction trials in chronic renal patients associated high hemoglobin targets and therefore higher ESA doses with mortality [29,30] or cardiovascular events [31,32]. In our study in HD patients with Hb level <11 g/dL, mortality rate was also increasing with increasing ESA doses. Only in patients with the lowest ESA dose mortality rate did not further decrease, as also previously published [33]. The patients with a high ESA dose and adequate Hb response in our study generally had a higher mortality rate as well, but a dose dependent effect was not consistently shown. Some other studies reported no harmful effect of high ESA dose [34-36].

In our cohort of patients ESA resistance was not only associated with cardiovascular mortality but also with non-cardiovascular mortality. A remarkable finding since the hypothesized mechanism through which high ESA dose would increase mortality, namely elevated arterial blood pressure, altered endothelial function and prothrombotic effects [37], would mainly result in cardiovascular causes of mortality. ESA resistance comprises more than just high ESA dose and is linked to malnutrition, inflammation, low iron stores, hyperparathyroidism, and other co-morbid conditions with increased mortality risk [38-41]. It may therefore be regarded as an indicator for disease severity, but even after adjustment for a wide range of these confounders ESA resistance is still associated with mortality. Thus the inability to achieve a proper hematopoietic response seems to be the best marker of worse prognosis. Secondary analyses of anemia correction trials also confirm this association between ESA resistance and mortality [12,42], but the causal pathway is not yet fully understood.

Some considerations are important in the interpretation of our results. Despite adjustment for a wide range of confounders, residual confounding cannot be excluded. Although our cohort comprised incident instead of prevalent dialysis patients, some potential survivor bias might be left because only patients that survived 6 months on dialysis were included in the present analyses (1474 patients). Our patients were not incident ESA users and changes in Hb, ESA dose or dialysis modality after baseline were not taken into account. Information on route of ESA administration, type of ESA, iron management and iron status besides ferritin was not available. Further investigation is needed to explore the role of these factors.

## Conclusions

In our cohort of incident dialysis patients, we showed that ESA resistance, defined by an above median ESA dose and Hb <11 g/dL, was associated with increased mortality in HD and PD patients. This strengthens the need to investigate and treat causes of ESA resistance not only in HD, but also in PD patients. Additional studies are needed to disentangle the effect of ESA resistance, high ESA doses and Hb level on mortality.

## Abbreviations

CI: Confidence interval; CRP: C-reactive protein; ERA-EDTA: European renal association- European dialysis and transplant association; ERI: Erythropoietin resistance index; ESA: Erythropoiesis-stimulating agent; Hb: Hemoglobin; HD: Hemodialysis; HR: Hazard ratio; IQR: Interquartile range; PD: Peritoneal dialysis; PTH: Parathyroid hormone; rGFR: Residual glomerular filtration rate; SD: Standard deviation; SGA: Subjective global assessment.

## Competing interests

The authors declare that they have no competing interests.

## Authors’ contributions

MS was involved in the design, analysis and interpretation of the data and drafted the manuscript. TH was involved in the design, analysis and interpretation of the data. JR, IO and MM were involved in the analysis and interpretation of the data. FD and RK were involved in the conception and design of the study, data acquisition and analysis and interpretation. All authors revised the article for important intellectual content and approved the final version of the manuscript.

## Pre-publication history

The pre-publication history for this paper can be accessed here:

http://www.biomedcentral.com/1471-2369/14/200/prepub
